# In Vitro Cultivation for *Glugea plecoglossi* (Microsporidia) Isolated from Ayu (*Plecoglossus altivelis*)

**DOI:** 10.3390/microorganisms12030522

**Published:** 2024-03-05

**Authors:** Guizong Xu, Zengyi Zhang, Qianjin Zhou, Mingyan Song, Guanjun Yang, Jinwei Kang, Zhongjie Xu, Fangjie Chen, Jiong Chen

**Affiliations:** 1 State Key Laboratory for Managing Biotic and Chemical Threats to the Quality and Safety of Agro-Products, Ningbo University, Ningbo 315211, China; xuguizong@163.com (G.X.); xuzhongjie1002@163.com (Z.X.); chenfangjiegroup@163.com (F.C.); 2 School of Marine Sciences, Ningbo University, Ningbo 315832, China; zhangzyi0320@163.com (Z.Z.); qq1052457580@163.com (M.S.); yangguanjun@nbu.edu.cn (G.Y.); hhswytzslzcdsh@163.com (J.K.); 3 Key Laboratory of Aquacultral Biotechnology Ministry of Education, Ningbo University, Ningbo 315211, China

**Keywords:** *Glugea plecoglossi*, *Plecoglossus altivelis*, cell culture, inflammatory cytokines, microsporidia

## Abstract

*Glugea plecoglossi* is an obligate intracellular microsporidium, which poses a significant threat to ayu (*Plecoglossus altivelis*). In vitro cultivation models are invaluable tools for investigating intracellular microorganisms, including *G. plecoglossil*. In this study, we attempted to in vitro cultivate *G. plecoglossi* using primary cultures derived from ayu monocytes/macrophages (MO/MΦ), a murine-derived macrophage cell line RAW264.7, and the epithelioma papulosum cyprini (EPC) cell line. The results demonstrated that MO/MΦ infected with spores exhibited a pronounced immune response which was presented by rapidly high expression levels of inflammatory cytokines, such as *PaIL-1β*, *PaTNF-α*, *PaIL-10*, and *PaTGF-β*, and detached within 96 h post-infection (hpi). Infected RAW264.7 cells remained capable of stable passage yet exhibited cellular deformation with a decrease in intracellular spores occurring around 8 days post-infection (dpi). In contrast, EPC cells promised a substantial parasite population, and the cytokine expression levels returned to normal by 8 dpi. In addition, *G. plecoglossi* spores recovered from EPC cells could infect young ayu, suggesting that EPC cells might be used as an in vitro cultivation system for *G. plecoglossi*.

## 1. Introduction

Microsporidia are widespread obligate intracellular parasites that exist in almost all known animal taxa [1,2]. They are characterized by the production of infective spores which transmit the infective sporoplasm to the host cytoplasm or nucleoplasm by a unique complicated eversion apparatus-polar tube yet exhibit a closer phylogenetic affinity to fungi [3,4,5]. Over 160 species across 22 genera have been identified in fish [6,7], and new species are continuously emerging [8,9,10]. Many microsporidians pose a significant threat to pisciculture. For example, microsporidians such as *Enterospora epinepheli* have been linked to the mass mortality of hatchery-bred juvenile groupers (*Epinephelus* spp.) in China [11]. Another example is *Enterospora nucleophila*, which has been identified as the causative agent of an emaciative syndrome observed in farmed gilthead sea bream (*Sparus aurata*) in Spain [12].

*Glugea plecoglossi* is a typically xenomas-forming microsporidium obligate to ayu (*Plecoglossus altivelis*) under natural infection conditions. This parasite was widely distributed in cultured ayu in China, which could lead to a rough and uneven appearance of the host [13]. However, research on this microsporidium is quite limited with sporadic studies primarily focused on identifying invasion-related cellular components (such as lectin-active components from the parasite and the host-derived rhamnose-binding lectin) [14,15] and the immunological events (such as phagocytosis and respiratory burst of macrophage) triggered by *G. plecoglossi* infection [14,16]. The life cycle, host–parasite interactions, and other related biological events of this microsporidium are still poorly understood.

In vitro cell culture has proven to be an invaluable tool for microsporidian-related research, including investigations into the life cycle and host–parasite interactions, which are attributed to their obligate intracellular parasitism. However, the majority of cell culture models have predominantly focused on microsporidians that infect arthropods and mammals. Few studies have delved into cell culture systems for fish-infecting microsporidians, which is possibly due to their host specificity and the unique characteristics of xenoma formation. Some limited yet effective attempts have been made for the in vitro cultivation of fish-infecting microsporidia, which are categorized into several scenarios. For example, short-term primary cultures derived from fish-specific tissues (such as the kidney) were used to demonstrate key cellular activity like the phagocytic capability of the macrophage [14]. Middle-term primary cultures or cell lines derived from fish allowed the observation of the transmission of infective cytoplasm into host cells by polar tubes, subsequent spores development, and even xenoma formation [17,18,19,20]. Some non-fish-derived cell lines were observed to permit limited intracellular spore proliferation of fish-infecting microsporidia [21,22].

In this study, we assessed the feasibility of in vitro cultivation of *G. plecoglossi* using primary cultures derived from ayu monocytes/macrophages (MO/MΦ), the murine-derived macrophage cell line RAW264.7, and the epithelioma papulosum cyprini (EPC) cell line. We documented the temporal patterns of spore entry into various cells and analyzed the dynamic changes of inflammatory cytokines during a certain period of infection. The objective is to establish a cell culture system for the in vitro cultivation of *G. plecoglossi*.

## 2. Materials and Methods

### 2.1. Fish Maintenance

The ayu used in this study were purchased from a commercial farm in Ninghai Country, Ningbo City, China. Healthy ayu, weighing approximately 20 g, were kept in freshwater tanks at 20–22 °C in a recirculating system with filtered water and acclimatized to laboratory conditions. Before the experiment, they were confirmed to be *G. plecoglossi* free using a loop-mediated isothermal amplification (LAMP) assay as described by Kang et al. [23]. All experiments were performed according to the Experimental Animal Management Law of China and approved by the Animal Ethics and Welfare Committee (AEWC) of Ningbo University (No. 11102).

### 2.2. G. plecoglossi Spores Purification

Xenomas of *G. plecoglossi* were stripped out of naturally infected ayu, and the spores were purified using a modified version of the method described by Lee et al. [24]. After two separation steps, the spore pellets were resuspended in a mixture of Percoll (Solarbio, Beijing, China) and sterile PBS in a 1:1 ratio and centrifuged at 1800× *g* for 30 min. The spores were maintained in sterile PBS containing specific antibiotics (100 U/mL penicillin, 100 μg/mL streptomycin, and 2.5 ng/mL amphotericin B (Solarbio, Beijing, China)). Purified spores were identified by LAMP and stored at 4 °C until use.

### 2.3. Culture of Primary MO/MΦ, EPC Cells and RAW264.7 Cells

MO/MΦ were isolated and cultured as previously described [25]. Briefly, the leukocyte-enriched fractions derived from the ayu head kidney were separated from the interface between the Ficoll and medium phases using Ficoll–Hypaque PREMIUM (1.077 g/mL) (GE Healthcare, South Plainfield, NJ, USA) according to the manufacturer’s instructions. Cells were cultured in RPMI 1640 medium (Invitrogen, Shanghai, China) for 12 h supplemented with 2% fetal bovine serum (FBS) (Invitrogen, Shanghai, China), penicillin (100 U/mL) and streptomycin (100 μg/mL) (Solarbio, Beijing, China). Non-adherent cells were gently pipetted off. The adherent cells were gently rinsed twice with sterile PBS (PH 7.4) to remove the remaining non-adherent cells and then incubated in complete medium (RPMI 1640, 5% ayu serum, 5% fetal bovine serum, 100 U/mL penicillin, and 100 μg/mL streptomycin) at 25 °C with 5% CO_2_. Following this cell separation procedure, over 96% of adherent cells were proved to be MO/MΦ [25].

The EPC cell line was kindly gifted by Prof. Ling-Bing Zeng (Yangtze River Fisheries Research Institute, Wuhan, China) and cultured in Medium 199 (Sparkjade, Qingdao, Shandong, China) supplemented with 10% fetal bovine serum, penicillin (100 U/mL), and streptomycin (100 μg/mL) at 25 °C for generation in a 5% CO_2_ incubator. The RAW264.7 cell line was provided by Dr. Li Nie (Ningbo University, Ningbo, China) and cultured in Dulbecco’s modified eagle medium (DMEM) (Invitrogen, Shanghai, China) supplemented with 10% fetal bovine serum, penicillin (100 U/mL), and streptomycin (100 μg/mL) at 37 °C for generation in a 5% CO_2_ incubator.

### 2.4. In Vitro Infection of Primary Cultures and Cell Lines Using G. plecoglossi Spores

On the day preceding the inoculation, ayu MO/MΦ were isolated and cultured in complete medium at a concentration of 2 × 10^7^ cells/mL at 25 °C, following the protocol outlined in Section 2.3. Meanwhile, both the EPC and RAW264.7 cell lines were seeded at a concentration of 2 × 10^6^ cells/mL and cultured at 25 °C. In the preliminary experiments, primary cultures or cell lines were infected with the spores at different multiplicity of infection (MOI) values of 1, 10, and 100. And it was found that infection with spores at an MOI of 10 yielded easily observable and recordable infection manifestations. Therefore, spore suspension at a concentration of 2 × 10^7^ cells/mL (an MOI of 10) was added to the pre-cultured primary cells or cell lines. Both MO/MΦ and the RAW264.7 cell were observed at 0.5, 2, 4, 8, 24, 96, and 192 h post-infection (hpi). Meanwhile, the EPC cells were continuously observed at 2, 3, 4, 5, 6 and 8 d post-infection (dpi). At 3 dpi, the cells were subjected to a new medium replacement, and the suspended spores present in the culture medium were washed away. The cells were viewed using the inverted fluorescence microscope (Nikon eclipse Ti-U, Tokyo, Japan) at a magnification of ×400.

### 2.5. Cell Staining

MO/MΦ, RAW264.7 and EPC were cultured on cover slips using the described culture conditions in Section 2.3 and were infected with the spores at an MOI of 10. The cells were harvested at 8 hpi, 8 hpi, and 6 dpi, respectively. The harvested cells were stained with Dil Stain (10 μM) (Beyotime, Shanghai, China) for 20 min, washed twice in PBS, and fixed with 4% paraformaldehyde (Solarbio, Beijing, China) for 20 min, which was followed by staining with DAPI (1 mg/mL) (Beyotime, Shanghai, China) or Calcofluor white M2R (10 μM) (Sigma-Aldrich, St. Louis, MO, USA). The slides were mounted with an anti-fluorescence quenching agent (Solarbio, Beijing, China) and observed using the confocal laser-scanning microscope Zeiss LSM 700 (Carl Zeiss, Jena, Germany) at a magnification of ×640.

### 2.6. Observation of G. plecoglossi Spores in Primary Cell and Cell Lines Using Transmission Electron Microscopy (TEM)

MO/MΦ, RAW264.7 and EPC cells were cultured on cover slips and infected using the spores at an MOI of 10 as described in Section 2.3. The spores infecting MO/MΦ, RAW264.7 and EPC cells were harvested at 24, 24 and 96 hpi and fixed with 5% glutaraldehyde (Sigma-Aldrich, St. Louis, MO, USA) for 48 h at 4 °C, which was intended for transmission electron microscopy observation. Cells were resuspended and washed three times using 0.1 M phosphate buffer (pH 7.4). After centrifugation, the precipitation was suspended with forceps and pre-embedded in 1% agarose. The agarose blocks were post-fixed with 1% OsO_4_ (Ted Pella, Inc., Redding, CA, USA) in 0.1 M phosphate buffer (pH 7.4) for 2 h at room temperature. After rinsing three times, the cells were dehydrated using a concentration gradient of alcohol and 100% acetone and embedded in resin (Servicebio, Wuhan, China). The ultrathin sections were cut and underwent 2% uranium acetate-saturated alcohol solution, avoiding light staining for 8 min, and 2.6% lead citrate, avoiding CO_2_ staining for 8 min, and then observed under TEM (HT7800/HT7700, Hitachi, Tokyo, Japan).

### 2.7. Cytokines Detection Using Real-Time Fluorescent Quantitative PCR

MO/MΦ, RAW264.7 and EPC were cultured in cell culture dishes and infected with the spores at an MOI of 10. MO/MΦ were harvested at 4, 12, 24, and 96 hpi, while RAW264.7 cells were harvested at 12, 24, 96, and 192 hpi. Meanwhile, EPC cells were harvested at 24, 72, 120, and 192 hpi. Three replicates were prepared for each sample. The expression level of cytokines, that is *IL-1β*, *TNF-α*, *IL-10* and *TGF-β*, was determined using real-time fluorescent quantitative PCR (real-time qPCR).

The total RNA was extracted from the cells using RNAiso reagent (TaKaRa, Dalian, China), and first-strand cDNA was synthesized using AMV reverse transcriptase (TaKaRa, Dalian, China) [26]. The qRT-PCR was performed on a QuantStudio™3 Real-Time PCR System (Thermo Fisher Scientific Inc., Waltham, MA, USA) using FastStart Essential DNA Green Master (Roche Molecular Diagnostics, Basel, Switzerland). The relative expression of the selected cytokines was calculated using the 2^−ΔΔCT^ method and normalized against the stable reference gene. All the primers used are listed in Table 1.

### 2.8. Re-Infection of Both Cell Lines and In Vivo Infection of Ayu Using EPC-Cultured G. plecoglossi Spores

Recovered EPC cells were infected with the spores for 5 days and centrifuged at 1100× *g* for 10 min. The cell pellets were suspended in sterile PBS and kept at 4 °C for 24 h. Then, the cells were pushed twice using a 1 mL syringe and subsequently mixed with Percoll. The mixture was centrifuged at 1800× *g* for 30 min. Following centrifugation, the resulting pellets were resuspended in the medium, and the spores were counted. The spore suspension was added to uninfected EPC cells and RAW264.7 cells at an MOI of 10. And 5 fish were intraperitoneally injected with EPC-cultured spores at a dose of 1 × 10^6^ cells each. Fish intraperitoneally injected with an equal volume of sterile PBS served as a negative control. At 21 dpi, fish were necropsied, and both liver and ovary were collected and fixed in 4% paraformaldehyde for 48 h. The paraffin tissue sections were prepared as described by Lu et al. [33]. Subsequently, the sections were stained with hematoxylin and eosin (H&E) (Solarbio, Beijing, China), which was followed by a secondary staining using M2R and observed under an upright fluorescence microscope (Nikon eclipse Ni-E, Tokyo, Japan).

## 3. Results

### 3.1. Light Microscopy Observation of Ayu MO/MΦ Infected with G. plecoglossi

**Figure 1:**
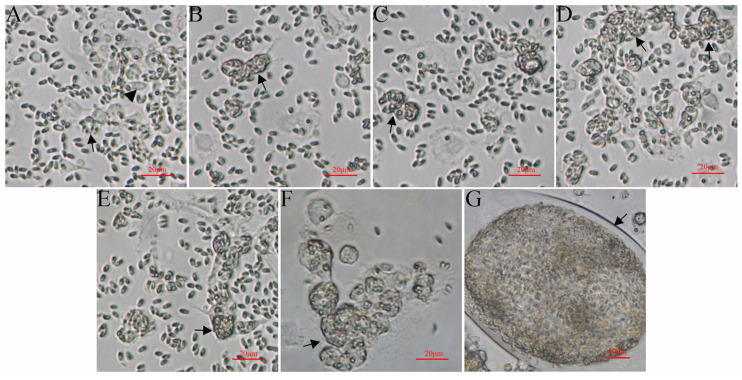
MO/MΦ infected with *G. plecoglossi* spores. (**A**–**G**): MO/MΦ infected with *G. plecoglossi* at an MOI of 10 at 0.5, 2, 4, 8, 24, 96, and 192 h, respectively. The cells were photographed using an inverted fluorescence microscope at a magnification of ×400. Arrow, MO/MΦ infected with *G. plecoglossi* spores; arrowhead, MO/MΦ without spores.

**Figure 2:**
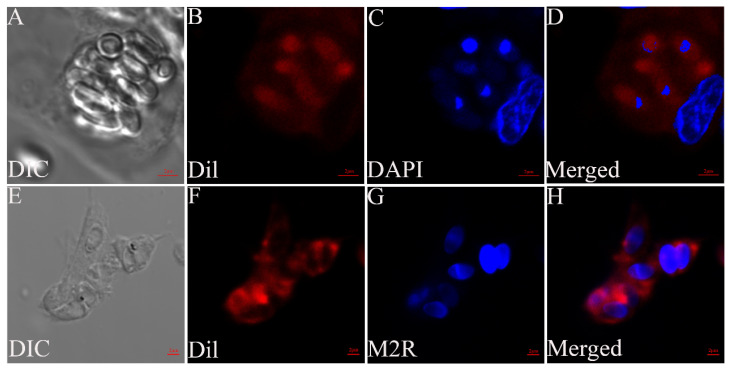
The infected MO/MΦ with *G. plecoglossi* spores stained with DAPI/Dil and M2R/Dil. Differential interference contrast (DIC) micrographs of MO/MΦ infected with *G. plecoglossi* spores (**A**,**E**). Dil-stained MO/MΦ membrane (red) (**B**,**F**). DAPI-stained nuclei (blue) (**C**), M2R-labeled spores (blue) (**G**), and merged images (**D**,**H**) are shown. Scale bar: 2 μm.

The MO/MΦ were infected with *G. plecoglossi* spores at an MOI of 10. The results showed that *G. plecoglossi* appeared in MO/MΦ at 0.5 hpi (Figure 1A). From 2 to 24 hpi, the number of *G. plecoglossi* in the cells rapidly increased (Figure 1B–E). At 8 hpi, infected MO/MΦ began to cluster (Figure 1D), and at 96 hpi, massive infected cells clustered together and exhibited pronounced protrusion (Figure 1F). At 192 hpi, a large number of infected cells with pronounced protrusion detached, and a portion of infected cells formed giant clusters or aggregates, which were filled with *G. plecoglossi* (Figure 1G). MO/MΦ infected with spores at 8 h were stained using two different compound dyes, DAPI and Dil (Figure 2A–D), as well as M2R and Dil (Figure 2E–H). This allowed for a clear observation that the spores structure of *G. plecoglossi* could be stained blue with DAPI, while M2R specifically stained the spores blue. Through this staining strategy, we could definitively observe the presence of multiple spores in MO/MΦ (Figure 2).

### 3.2. Light Microscopy Observation of RAW264.7 Cells Infected with G. plecoglossi

**Figure 3:**
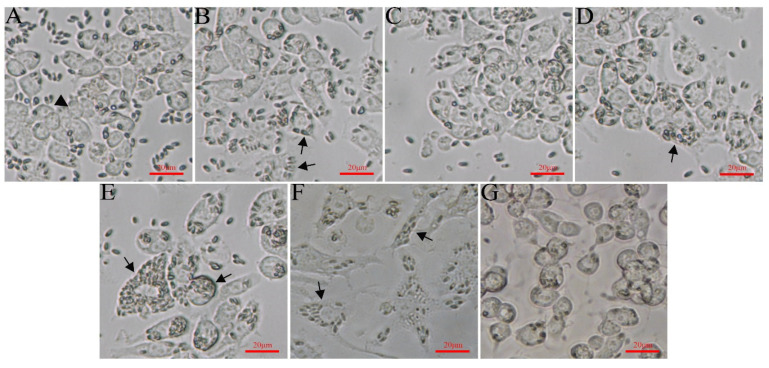
RAW264.7 cells infected with *G. plecoglossi* spores. (**A**–**G**): RAW264.7 cells infected with *G. plecoglossi* at an MOI of 10 at 0.5, 2, 4, 8, 24, 96, and 192 hpi. The cells were photographed using an inverted fluorescence microscope at a magnification of ×400. Arrow, RAW264.7 cells infected with *G. plecoglossi* spores; arrowhead, the RAW264.7 cell without spores.

**Figure 4:**
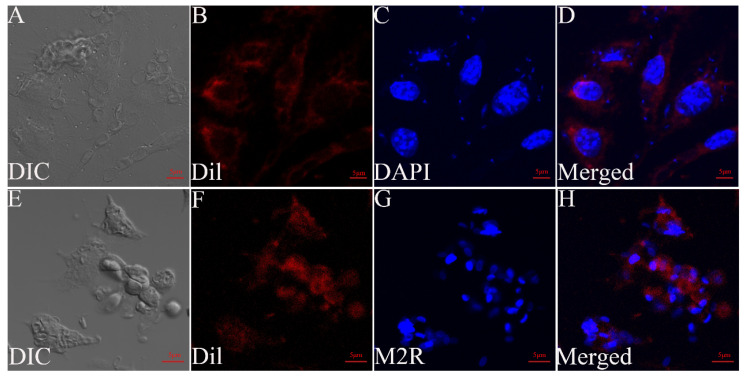
The infected RAW264.7 cells with *G. plecoglossi* spores stained with DAPI/Dil and M2R/Dil. DIC micrographs of RAW264.7 cells infected with *G. plecoglossi* spores (**A**,**E**). Dil-stained membrane (red) (**B**,**F**). DAPI-stained nuclei (blue) (**C**), M2R-labeled spores (blue) (**G**), and merged images (**D**,**H**) are shown. Scale bar: 5 μm.

The RAW264.7 cells were infected with *G. plecoglossi* spores at an MOI of 10. The results indicated that *G. plecoglossi* was observed in RAW264.7 cells at 2 hpi (Figure 3A,B) and gradually increased until 24 hpi (Figure 3C–E). By 96 hpi, infected RAW264.7 cells exhibited elongation and morphological polymorphism (Figure 3E,F), which was accompanied by a gradual decrease in the number of *G. plecoglossi* spores. At 192 hpi, the spores within RAW264.7 cells became scarce, and the cells’ morphology returned to normal (Figure 3G). The DAPI/Dil staining showed the deformation of infected RAW264.7 cells at 24 hpi and the internal *G. plecoglossi* spores (Figure 4A–D). Multiple spores were also observed in the infected RAW264.7 cells by M2R/Dil staining (Figure 4E–H).

### 3.3. Light Microscopy Observation of EPC Cells Infected with G. plecoglossi

**Figure 5:**
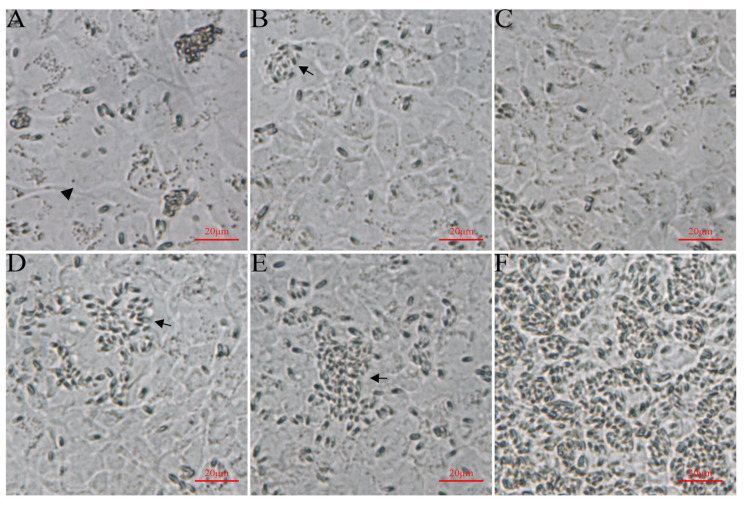
EPC cells infected with *G. plecoglossi* spores. (**A**–**F**): EPC cells infected with *G. plecoglossi* at an MOI of 10 at 2, 3, 4, 5, 6, 8 dpi. The cells were photographed using an inverted fluorescence microscope at a magnification of ×400. Arrow, EPC cells infected with *G. plecoglossi* spores; arrowhead, the EPC cell without spores.

**Figure 6:**
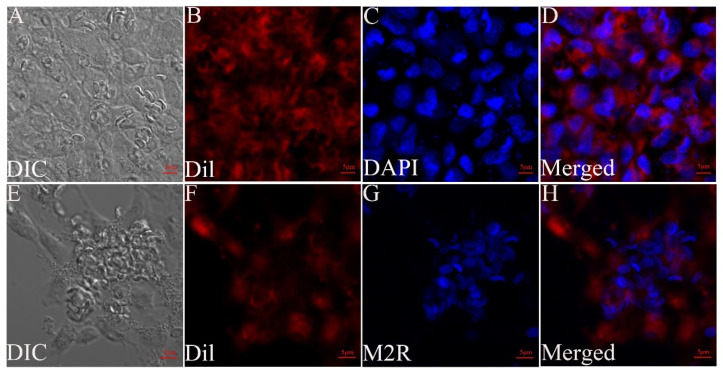
EPC cells infected with *G. plecoglossi* spores. The infected EPC cells with *G. plecoglossi* spores stained with DAPI/Dil and M2R/Dil. DIC micrographs of EPC cells infected with *G. plecoglossi* spores (**A**,**E**). Dil-stained membrane (red) (**B**,**F**). DAPI-stained nuclei (blue) (**C**), M2R-labeled spores (blue) (**G**), and merged images (**D**,**H**) are shown. Scale bar: 5 μm.

EPC cells were infected with *G. plecoglossi* spores at an MOI of 10. A small number of spores were observed inside EPC cells at 3 dpi (Figure 5A,B). From 4 dpi, the spores gradually increased, and they almost filled all EPC cells at 8 dpi (Figure 5C–F). Both DAPI/Dil and M2R/Dil staining showed massive spores within EPC cells (Figure 6).

### 3.4. TEM Observation of G. plecoglossi Spores in Primary Cell and Cell Lines

**Figure 7:**
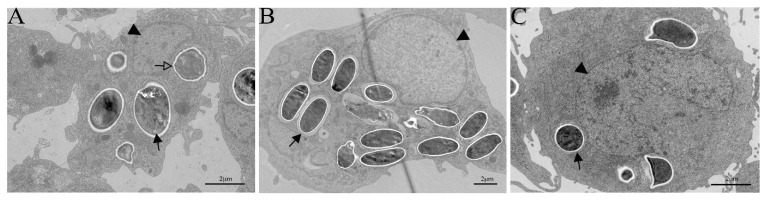
Ultrastructure of spore stages of *G. plecoglossi* in primary cultures and cell lines. (**A**) Infected ayu MO/MΦ, (**B**) RAW264.7 cells, and (**C**) EPC cells. Arrows showed the mature spores of *G. plecoglossi*; arrowhead indicated the host cell nucleus; hollow arrow demonstrated the spores with a gear-like spore wall in MO/MΦ (**A**) or under degradation in RAW264.7 cells (**B**). Scale bar = 5 µm.

TEM revealed varying quantities of *G. plecoglossi* spores present within the cellular milieu. The spores were evident in the cell cytoplasm of infected ayu MO/MΦ, RAW264.7, and EPC cells (Figure 7). No other developmental stages of the microsporidian were apparently observed in the MO/MΦ and both cell lines. Notably, in MO/MΦ at 24 hpi, some spores displayed gear-like structures on their walls, which was indicative of damage inflicted by the host cell (Figure 7A). Additionally, a degradation of spores was observed in RAW264.7 cells at 24 hpi (Figure 7B). In infected EPC cells, a distinctive structural change was observed, which was characterized by the enlargement and elongation of the cell nucleus (Figure 7C).

### 3.5. The Cytokines Expression in MO/MΦ, RAW264.7, and EPC Cells upon G. plecoglossi Infection

**Figure 8:**
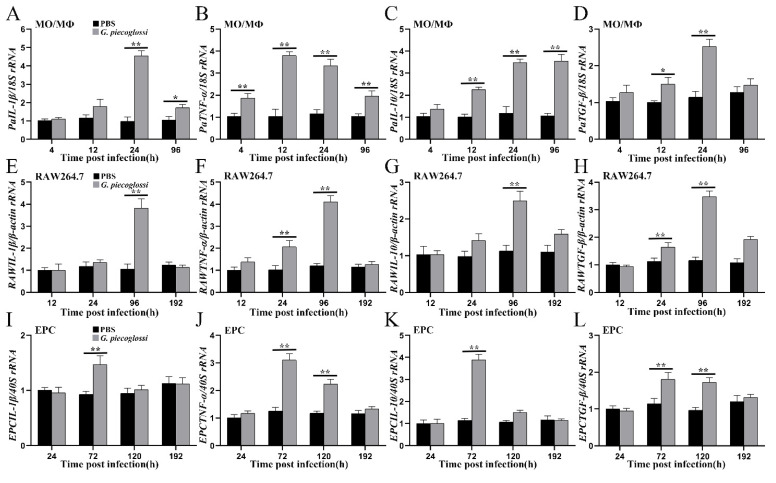
The mRNA levels of cytokines in MO/MΦ, EPC, and RAW264.7 cells upon *G. plecoglossi* infection. MO/MΦ, EPC and RAW264.7 cells were treated with *G. plecoglossi* at an MOI of 10. The mRNA levels of *IL-1β* (**A**,**E**,**I**), *TNF-α* (**B**,**F**,**J**), *IL-10* (**C**,**G**,**K**), and *TGF-β* (**D**,**H**,**L**) in MO/MΦ, EPC and RAW264.7 cells were evaluated by real-time qPCR. *, *p* < 0.05 as compared to PBS treated group; **, *p* < 0.01 as compared to PBS treated group.

The results demonstrated that spore infection caused notable kinetic alterations in cytokines within MO/MΦ, such as *PaIL-1β*, *PaTNF-α*, *PaIL-10* and *PaTGF-β* (Figure 8A–D). Specifically, the expression of *PaIL-1β*, *PaTNF-α*, and *PaTGF-β* initially increased and then decreased after infection. At 24 hpi, *PaIL-1β* expression significantly increased (*p* < 0.01). Even at 96 hpi, despite a declining trend, the expression level remained significantly higher than that in the control group (*p* < 0.05). *PaTNF-α* expression significantly increased (*p* < 0.01) at 4 hpi, peaked at 12 hpi, and remained significantly higher than that in the control group at 96 hpi (*p* < 0.01). *PaTGF-β* expression peaked at 24 hpi (*p* < 0.01) and returned to a level comparable to the control group. The expression level of *PaIL-10* significantly increased at 12 hpi (*p* < 0.01), followed by a continuous rise, maintaining elevated levels from 24 to 96 hpi (*p* < 0.01).

In RAW264.7 cells, within 12 to 192 hpi, the expression of cytokines, namely *RAWIL-1β*, *RAWTNF-α*, *RAWIL-10* and *RAWTGF-β*, exhibited a trend of initial increase followed by a decrease (Figure 8E–H). The expression levels of these four cytokines peaked at 96 hpi (*p* < 0.01). At 192 hpi, except for the expression of *RAWTGF-β*, which remained higher than that in the control group (*p* < 0.01), the expression levels of the other three cytokines returned to levels comparable to those in the control group. In contrast, in EPC cells, the expression levels of these four cytokines peaked at 72 hpi (*p* < 0.01), and all of them returned to levels comparable to those in the control group at 192 hpi (Figure 8I–L).

### 3.6. The Infectivity of Spores Cultured in EPC on Cell Lines and Ayu

**Figure 9:**
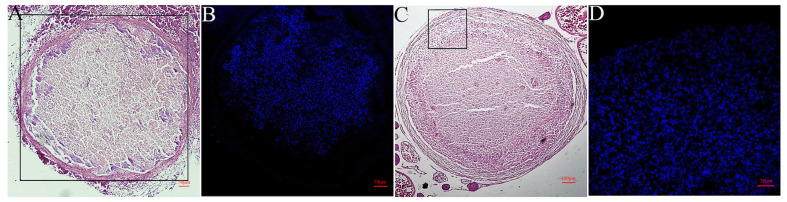
Spore observation from artificially infected ayu with EPC-cultured *G. plecoglossi* spores. The liver and ovaries were collected from infected ayu with EPC-cultured *G. plecoglossi* spores and stained with H&E (**A**,**C**) and M2R (**B**,**D**). Small spherical cysts resembling xenomas were found in the liver (**A**,**B**) and ovaries (**C**,**D**).

*G. plecoglossi* spores harvested from EPC cells were capable of re-infecting cell lines, namely EPC and RAW264.7 (data not shown). Meanwhile, the spores of *G. plecoglossi* were detectable in the liver and ovaries of ayu that were intraperitoneally administered with EPC-cultured spores at 21 dpi (Figure 9). Furthermore, small spherical cysts resembling xenomas were identified in the liver and ovaries, confirming their content to be filled with *G. plecoglossi* (Figure 9B,D).

## 4. Discussion

Cell cultures are proven invaluable tools for investigating all biological processes of intracellular microorganisms, determining, to a certain extent, the depth and breadth of such research. The lack of well-established cell culture models capable of recapitulating the life cycle of fish microsporidia in vitro hinders research progress in this field. Many attempts have been made to study the cultivation of fish microsporidia within primary cultures and cell lines [18,20,21,34]. In this study, we conducted an exploration into the cultivation of *G. plecoglossi* within ayu MO/MΦ, RAW264.7 cells, and EPC cells, respectively. *G. plecoglossi* spores were capable of rapidly infiltrating MO/MΦ and RAW264.7 cells. In MO/MΦ, spores invasion led to substantial detachment and cell death within 96 hpi. In RAW264.7 cells, the spore count was under a precipitous reduction within 8 dpi. In contrast, a sustained escalation was observed in spore quantity within EPC cells within 8 dpi. In addition, we revealed the dynamic changes of four common cytokines in the three types of cells during spore infection, and we also confirmed the specific utility of M2R in staining microsporidian spores.

Microsporidia, including species derived from fish, often exhibit a tendency to infect one or several closely related hosts [35]. This host specificity poses challenges in identifying primary cultures or cell lines that can be universally applicable for the in vitro cultivation of various microsporidia. Xenoma development further complicates the in vitro culture of fish microsporidia compared to their mammalian counterparts [36]. Attempts were made to develop primary cultures of immune cells, including macrophages, neutrophils, and lymphocytes, from the natural hosts for short-term cultivation (e.g., within 48 h) of microsporidia [14,18]. A relatively long-term in vitro cultivation of microsporidia was succeeded in several fish-derived cell lines. A Chinook salmon (*Oncorhynchus tshawytscha*) embryo cell line CHSE-214 was attempted to cultivate *Glugea* sp. isolated from greater sand eels (*Hyperoplus lanceolatus*) [17]. The kidney-derived mononuclear leukocytes and stromal epithelial cells from Chinook salmon were used to develop a relatively long-term culture of *Enterocytozoon salmonis* [18]. *Anncaliia algerae*, an aquatic microsporidium commonly infecting mosquitoes, was capable of growth in several fish cell lines, namely GFSK-S1 and GFB3C-W1 from goldfish, ZEB2J from zebrafish, and FHMT-W1 from fathead minnow [21]. Interestingly, McConnachie et al. revealed that *Loma salmonae* could develop xenomas within a rainbow trout gill epithelial cell line [19]. Meanwhile, xenomas development of *Loma morhua* was also observed in a continuous cell line from larval Atlantic cod (*Gadus morhua*) [20]. In addition, fish microsporidia seem to have the capacity of growth on the cell culture systems that differ in the class or group of apparent natural hosts [18,21,22]. For example, *Heterosporis saurida* infecting lizardfish, *Saurida undosquamis*, could not grow on fish-derived cell lines, such as common carp brain (CCB-816), EPC, fathead minnow epithelial (FHM-57), rainbow trout gonad (RTG-2), bluegill fry (BF-2) and CHSE-214 but could grow on rabbit kidney epithelial (RK-13) [22]. *A. algerae* could also be grown on RK-13 [21]. In this study, we observed the rapid appearance of *G. plecoglossi* in MO/MΦ isolated from its natural host within 0.5 hpi. This triggered a robust response in the primary cultures, characterized by cell clustering and pronounced protrusion, with the majority detaching at 8 dpi, making it challenging to cultivate *G. plecoglossi* in MO/MΦ for an extended period (Figure 1). The short-term cultivation system allowed exploration of only limited immunological events during the early stages or acute phase of infection [14,16]. *G. plecoglossi* could also appear in RAW264.7 cells within 0.5 hpi, and intracellular spore accumulation induced cell deformation and detachment within 96 hpi. However, through subsequent cell passaging and subculturing, the spores were gradually eliminated, leading to the recovery of normal cellular morphology by 8 dpi (Figure 3). *G. plecoglossi* only sporadically appeared in EPC cells until 3 dpi, and they almost filled all EPC cells at 8 dpi (Figure 5). Spores collected from EPC cells successfully infected young ayu individuals, indicating that EPC cells can serve as an in vitro cultivation system for the mass production of infective spores of *G. plecoglossi* (Figure 9).

Xenoma is a unique host–parasite complex derived from the host cell, characterized by hypertrophic growth, which creates a distinct environment conducive to parasite proliferation [6,37]. Xenoma development in many fish microsporidia, such as *Glugea* spp. and *Loma* spp., induces a comprehensive and viable restructuring of the infected host cell, adding complexity to the understanding of the host immune response against microsporidia infection, which still remains a mystery [1,3]. Kim et al. described that ayu MO/MΦ could phagocytize *G. plecoglossi* spores via ConA-reactive glycoprotein-mediated recognition [14]; these spores probably established infection by modulating the host’s phagocytic response [16]. Leiro et al. demonstrated that *Tetramicra brevifilum* spores partially suppressed the respiratory burst response of turbot (*Scophthalmus maximus*) phagocytes [38]. *Enterospora nucleophila* infection induced macrophage aggregates containing spores in intestinal submucosa to develop into granulomata with necrotic areas containing parasite remnants, and this local submucosa was abundant with IgM^+^ and IgT^+^ cells in gilthead sea bream (*Sparus aurata*) [39]. However, the molecular immunological events within host cells induced by fish microsporidia remain virtually unknown. In mammals, the pro-inflammatory (Th1) cytokines, like interferon-γ (IFN-γ), tumor necrosis factor-α (TNF-α), and interleukin-12 (IL-12) were observed to increase rapidly against taxa belonging to the genus *Encephalitozoon* including so-called *Septata* infection [40]. IFN-γ and IL-12 could activate CD8^+^ cytotoxic T lymphocytes (CTLs), which played a crucial role in the protection against *E. cuniculi* [41]. Toll-like receptor 2 (TLR2) and TLR4 have been implicated in recognizing taxa belonging to the genus *Encephalitozoon* including so-called *Septata* by binding to glycans on the surface of microsporidia, such as phospholipomannans and O-linked mannans [1,2]. This recognition probably activated intracellular pathways, including NF-κB, and the secretion of chemokines [1,2]. In the present study, several pro-inflammatory and anti-inflammatory cytokines, such as *PaTNF-α*, *PaIL-1β*, *PaIL-10*, *PaTGF-β*, exhibited a significant increase in the early stages of *G. plecoglossi* infection of ayu MO/MΦ (4 or 12 hpi), which implied a robust immune response. By 24 to 96 hpi, the expression levels of several pro-inflammatory cytokines gradually returned to the usual status, while the anti-inflammatory cytokine like *PaIL-10* maintained a high level at 96 hpi (Figure 8). Upon *G. plecoglossi* infection of non-natural host cells RAW264.7 and EPC cells, these cytokines displayed a similar yet relatively delayed expression profile compared to MO/MΦ (Figure 8). For example, *RAWTGF-β* remained significantly upregulated in RAW264.7 cells at 192 hpi, while the expression of *EPCTNF-α* and *EPCTGF-β* markedly increased in EPC cells at 120 hpi (Figure 8).

Calcofluor White M2R could bind to chitin in the microsporidian spore wall and has been utilized to specifically label the sporogonic phase of microsporidia [42]. For instance, it has been employed in differentiating viable spores of *Encephalitozoon cuniculi* [43], distinguishing the intracellular cycle of *Nosema bombycis* using cells or tissue slices [44], and conducting immunofluorescence analysis of *Enterocytozoon hepatopenaei*-infected hepatopancreatic tissue [45]. In this study, M2R was applied to stain intracellular *G. plecoglossi*, demonstrating its capability to discriminate *G. plecoglossi* spores from MO/MΦ, RAW264.7, and EPC cells (Figure 2, Figure 4 and Figure 6). Additionally, M2R could distinguish the spores from tissue sections when combined with histopathological H&E staining (Figure 9). This approach has also been referenced in the identification of *E. hepatopenaei* in the hepatopancreas tissue of *Penaeus vannamei* [46].

## 5. Conclusions

We attempted the in vitro cultivation of *G. plecoglossi* using ayu MO/MΦ, a primary cell from the natural host, and observed that the majority of the infected MO/MΦ exhibited a pronounced immune response and detached within 96 hpi. In contrast, the mammalian macrophage cell line RAW264.7 remained capable of stable passage after infection, but the intracellular parasites became scarce around 8 dpi. EPC cells sustained a substantial parasite population and demonstrated a scenario where cytokine expression levels return to normal by 8 dpi. Additionally, *G. plecoglossi* spores recovered from EPC cells could infect young ayu, suggesting that EPC cells might serve as an in vitro cultivation system for *G. plecoglossi*. However, the suitability of EPC cells as an in vitro model for exploring the invasion mechanism of *G. plecoglossi* requires further discussion given that EPC cells are non-susceptible cells from a non-natural host for this microsporidium.

## Figures and Tables

**Table 1:** Oligonucleotide primer sequences used in this study.

Gene	GenBank No.	Primer	Nucleotide Sequence (5′→3′)	Resource
*PaTNF-α*	JP740414	PaTNF-αF	ACATGGGAGCTGTGTTCCTC	[25]
		PaTNF-αR	ACATGGGAGCTGTGTTCCTC	
*PaIL-1β*	HF543937	PaIL-1βF	TACCGGTTGGTACATCAGCA	
		PaIL-1βR	TGACGGTAAAGTTGGTGCAA	
*PaTGF-β*	JP742920	PaTGF-βF	CTGGAATGCCGAGAACAAAT	
		PaTGF-βR	CTGGAATGCCGAGAACAAAT	
*PaIL-10*	JP758157	PaIL-10F	TGCTGGTGGTGCTGTTTATGTGT	
		PaIL-10R	AAGGAGCAGCAGCGGTCAGAA	
*Pa18S rRNA*	FN646593	Pa18SF	GAATGTCTGCCCTATCAACT	
		Pa18SR	GATGTGGTAGCCGTTTCT	
*RAWTNF-α*	NM013693	RAWTNF-αF	CCTCACACTCAGATCATCTTCTC	[27]
		RAWTNF-αR	AGATCCATGCCGTTGGCCAG	
*RAWIL-1β*	NM008361	RAWIL-1βF	CTTTGAAGAAGAGCCCATCC	[28]
		RAWIL-1βR	TTTGTCGTTGCTTGGTTCTC	
*RAWTGF-β*	NM011577	RAWTGF-βF	CAAGGAGACGGAATACAGGG	[29]
		RAWTGF-βR	CGCACACAGCAGTTCTTCTC	
*RAWIL-10*	NM010548	RAWIL-10F	GCTCTTACTGACTGGCATGAG	[30]
		RAWIL-10R	CGCAGCTCTAGGAGCATGTG	
*RAWβ-actin*	NM007393	RAWβ-actinF	CCTAAGGCCAACCGTGAAAAG	[31]
		RAWβ-actinR	TCTTCATGGTGCTAGGAGCCA	
*EPCTNF-α*	JN412133	EPCTNF-αF	CAAGCAATTGGCGAGTGTGT	[32]
		EPCTNF-αR	CAGTTCCACTTTCCTGATTACTCTGA	
*EPCIL-1β*	AJ245635	EPCIL-1βF	AGACCAATCTCTACCTCGCTTGTAC	
		EPCIL-1βR	TTAATGGTGTTTAATGTTTCACTGATCTC	
*EPCTGF-β*	JN412135	EPCTGF-βF	TGTATAACAGCACTGTCGAGCTAAGC	
		EPCTGF-βR	TCCCTTCTCATTAGGATCTTCTACATC	
*EPCIL-10*	JN412134	EPCIL-10F	GATGTCACGTCATGGACGAGAT	
		EPCIL-10R	GGACTGGAAGTGGTTCTTCTGTACA	
*EPC40S*	JN412136	EPC40SF	TTTTGAGAAGAGGCATAAGAACATGT	
		EPC40SR	GTAACGATGTCACCAACAGTCACA	

## Data Availability

Data are contained within the article.
